# PRG4-Related Camptodactyly–Arthropathy–Coxa Vara–Pericarditis Syndrome Mimicking Juvenile Idiopathic Arthritis: A Case-Based Review

**DOI:** 10.3390/ijms27083534

**Published:** 2026-04-15

**Authors:** Nataliya Tkachenko, Cláudia Castelo Branco

**Affiliations:** 1 Department of Genetics, Hospital Divino Espirito Santo of Ponta Delgada, Av. D. Manuel I, 9500-782 Ponta Delgada, Azores, Portugal; claudia.ma.branco@azores.gov.pt; 2 Faculty of Sciences and Technology, University of the Azores (UAc), 9500-321 Ponta Delgada, Azores, Portugal

**Keywords:** juvenile idiopathic arthritis, CACP syndrome, PRG4, pediatric arthropathy, misdiagnosis

## Abstract

Juvenile idiopathic arthritis (JIA) represents the most common cause of chronic arthritis in childhood; however, not all early-onset arthropathies are inflammatory in origin. We report the case of a 4-year-old girl initially diagnosed with oligoarticular JIA and treated with methotrexate followed by a tumor necrosis factor inhibitor, without significant clinical improvement and despite persistently normal inflammatory markers. Clinical reassessment raised suspicion of a non-inflammatory arthropathy, supported by characteristic radiographic findings including metaphyseal flaring of the distal femora and proximal tibiae. Genetic analysis identified compound heterozygous pathogenic variants in the *PRG4* gene, confirming the diagnosis of camptodactyly–arthropathy–coxa vara–pericarditis (CACP) syndrome (OMIM #208250). PRG4 encodes lubricin, a mucin-like glycoprotein essential for boundary lubrication of articular cartilage and maintenance of synovial joint homeostasis. Loss-of-function variants disrupt joint lubrication, leading to mechanical synovial hyperplasia and chronic non-inflammatory joint effusion. This case highlights common diagnostic pitfalls in pediatric rheumatology and underscores the importance of considering genetic causes of chronic arthropathy when clinical and laboratory features are atypical for inflammatory disease. Early molecular diagnosis prevents unnecessary immunosuppressive therapy and enables appropriate multidisciplinary management.

## 1. Introduction

Juvenile idiopathic arthritis (JIA) represents the most frequent cause of chronic arthritis in children and is primarily defined by clinical and laboratory features of inflammation. In routine pediatric practice, persistent joint swelling in early childhood is often presumed to be inflammatory in origin, leading to early initiation of immunosuppressive therapy [1,2]. Although genetic susceptibility plays a role in certain JIA subtypes, most notably systemic JIA, in which associations with HLA-DRB1*11 and other MHC class II variants have been described [3], the presence of arthritis alone does not equate to immune-mediated disease.

However, a subset of pediatric arthropathies is non-inflammatory and genetically determined. Among these, camptodactyly–arthropathy–coxa vara–pericarditis (CACP) syndrome is a rare autosomal recessive disorder caused by pathogenic variants in the *PRG4* gene, which encodes lubricin, a glycoprotein essential for joint lubrication and synovial homeostasis [4,5,6]. Lubricin is a mucin-like glycoprotein secreted primarily by synovial fibroblasts and superficial zone chondrocytes. It forms a boundary lubrication layer at the cartilage surface, reducing friction between articulating joint surfaces and preventing mechanical wear. Experimental models have demonstrated that PRG4 deficiency leads to increased cartilage friction, synovial proliferation and accumulation of non-inflammatory joint effusion, providing a molecular explanation for the characteristic phenotype observed in CACP syndrome [7,8].

Due to overlapping clinical features, CACP syndrome is frequently misdiagnosed as juvenile idiopathic arthritis, particularly in early stages, leading to delays in appropriate diagnosis and management [1,9]. Several recent studies have reported cases of CACP syndrome initially misdiagnosed as juvenile idiopathic arthritis, leading to inappropriate use of immunosuppressive therapies [5,10]. In such cases, identification of pathogenic *PRG4* variants was essential to establish the correct diagnosis and avoid ineffective treatments [10].

Here, we present a case-based review illustrating how CACP syndrome can mimic juvenile idiopathic arthritis and highlight key clinical and radiological features that should prompt diagnostic reconsideration in pediatric practice [2,11]. In addition, this work underscores the growing importance of a multidisciplinary clinical approach in the evaluation and management of children with atypical or treatment-refractory arthropathies.

## 2. Case Description

A 4-year-old girl, the second child of young, healthy, non-consanguineous parents, was referred for genetics consultation for further evaluation of chronic joint swelling. The father has a history of migraine and asthma, and the mother is healthy. She has a 9-year-old sister with atopic eczema. There is no family history of autoimmune, neurological, or genetic disorders.

Pregnancy and perinatal history were unremarkable. She was born at term (39 weeks and 6 days of gestation) by cesarean section due to arrest of labor progression, with normal neonatal adaptation (Apgar scores of 10/10/10 at 1, 5, and 10 min, respectively). Birth parameters were within normal limits (weight 3600 g; length 50.5 cm; head circumference 36 cm). The neonatal period was uneventful, and universal neonatal hearing screening using otoacoustic emissions was normal, with bilateral pass results.

Growth parameters, head circumference, and global psychomotor development were appropriate for age. She achieved motor milestones on time, walking and running independently and climbing stairs. Fine motor and language development were normal. No feeding or sleep disturbances were reported, and vaccinations were up to date.

Her medical history was notable for plagiocephaly treated with physiotherapy, recurrent episodes of painful radial head subluxation since the first year of life, and trigger fingers affecting the second digit of the right hand and the first digit of the left hand, surgically corrected at 24 months of age (A1 pulley release), with residual flexion contracture of the third finger of the right hand.

From approximately 2 years of age, she developed progressive bilateral knee swelling, later involving the ankles. Importantly, this occurred without pain, morning stiffness, functional limitation, fever, or systemic symptoms, and there was no history of trauma. At 2 years and 3 months of age, she was referred for pediatric rheumatology evaluation due to suspected synovitis. Ultrasound examination of the knees and ankles revealed moderate synovitis with marked joint effusion in both knees, moderate involvement of the left tibiotalar joint and mild involvement of the right, as well as bilateral tenosynovitis of the peroneal and posterior tibial tendons. These findings were interpreted as oligoarthritis with features suggestive of an inflammatory process.

She was subsequently followed in Pediatric Rheumatology and diagnosed with Juvenile Idiopathic Arthritis (oligoarticular subtype). Antinuclear antibodies were negative, and uveitis was excluded. Treatment with methotrexate (10 mg/week; 15 mg/m^2^) was initiated, followed by adalimumab (20 mg every two weeks) due to poor response. Despite therapy, there was no meaningful clinical improvement. Moreover, treatment was associated with adverse effects, including recurrent respiratory infections and hepatic steatosis. Serial laboratory investigations consistently demonstrated normal or minimally elevated inflammatory markers (C-reactive protein and erythrocyte sedimentation rate), negative autoimmunity (antinuclear antibodies, rheumatoid factor, anti-CCP antibodies, HLA-B27 and HLA-DRB1*11), and negative infectious workup.

At 3 years and 6 months of age, the patient underwent arthrocentesis of both knees, yielding 23 mL from the right knee and 18 mL from the left. The synovial fluid was yellow-orange, slightly turbid, and of decreased viscosity. Cytological analysis revealed low cellularity (330 cells/mm^3^) with predominance of mononuclear cells, while biochemical analysis showed normal glucose and protein levels, with only a slight increase in lactate dehydrogenase. These findings were not consistent with a frankly inflammatory process.

**Figure 1:**
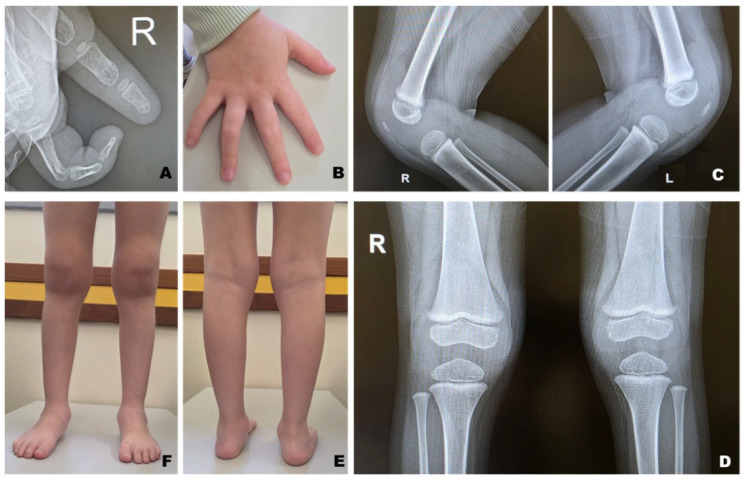
Clinical and radiological features of the patient with camptodactyly–arthropathy–coxa vara–pericarditis (CACP) syndrome. (**A**) Radiograph of the right hand showing residual camptodactyly of the index finger, with persistent flexion at the proximal interphalangeal joint and preserved bone structure, without erosions. (**B**) Clinical photograph of the hands demonstrating mild camptodactyly, more evident in the third finger of the right hand, associated with slender, tapered fingers, with no signs of local inflammation. (**C**) Lateral radiographs of both knees showing periarticular soft tissue enlargement consistent with joint effusion, without erosive changes. (**D**) Anteroposterior radiographs of both knees demonstrating metaphyseal widening of the distal femora and proximal tibiae, producing a characteristic Erlenmeyer flask–like deformity. (**E**) Posterior view of the lower limbs showing involvement of the knees, ankles, and feet, with flat feet and symmetrical hindfoot alignment. (**F**) Anterior view of the lower limbs demonstrating symmetrical, painless swelling of the knees and ankles, without external inflammatory signs.

Imaging studies demonstrated bilateral joint effusion and synovial hypertrophy. Plain radiographs of the knees revealed metaphyseal widening with an Erlenmeyer flask–like deformity of the distal femora and proximal tibiae, with preserved joint alignment and absence of erosive changes (Figure 1). Metabolic and lysosomal storage disorders, including Gaucher disease and mucopolysaccharidosis type I, were excluded.

Given the absence of systemic inflammation, lack of response to immunosuppression, history of trigger fingers and flat feet, and characteristic radiographic findings, a non-inflammatory genetic arthropathy was suspected. Genetic analysis identified compound heterozygosity for a pathogenic variant, c.2806_2810del p.(Lys936AspfsTer40), and a likely pathogenic variant, c.3787_3788del p.(Lys1263GlufsTer11), in the *PRG4* gene. The frameshift variant c.2806_2810del p.(Lys936AspfsTer40) is located in exon 7 of 13 of the *PRG4* gene (NM_005807.6) and results in a shift in the reading frame with the introduction of a premature stop codon, predicted to produce a truncated protein or lead to transcript degradation through the nonsense-mediated decay (NMD) pathway. Loss-of-function variants in *PRG4* are an established disease mechanism (CCID:005880). This variant (rs763025365) is classified as pathogenic in the ClinVar database (ID: 5651) and is reported in the population database gnomAD with an allele frequency of 0.000004338 in the general population. The variant has previously been described in the literature in homozygosity in two siblings with camptodactyly–arthropathy–coxa vara–pericarditis syndrome (CACP) (PMID: 10545950). To date, no functional studies have been reported to further clarify its biological effect. Based on the available evidence, the *PRG4* variant c.2806_2810del p.(Lys936AspfsTer40) is classified as pathogenic. The frameshift variant c.3787_3788del p.(Lys1263GlufsTer11) is located in exon 10 of 13 of the *PRG4* gene (NM_005807.6) and similarly results in a frameshift leading to a premature termination codon, predicted to produce a truncated protein or result in transcript degradation through the NMD pathway. Loss-of-function variants in *PRG4* are an established disease mechanism (CCID:005880). This variant has not been previously reported in ClinVar, the scientific literature, or the population database gnomAD. No functional studies are currently available to clarify its biological effect. Based on the available evidence, the *PRG4* variant c.3787_3788del p.(Lys1263GlufsTer11) is classified as likely pathogenic. Genetic analysis was performed using next-generation sequencing of a clinical exome panel, followed by variant confirmation and interpretation according to ACMG guidelines.

Considering the clinical, laboratory, radiological, and genetic findings described above, the diagnosis of CACP syndrome was established and the initial diagnosis of JIA was subsequently revised. Immunosuppressive therapy was therefore discontinued. The patient was referred for multidisciplinary follow-up, primarily focused on orthopedic evaluation and rehabilitative management.

## 3. Discussion

### 3.1. CACP Syndrome as a Mimicker of Juvenile Idiopathic Arthritis

The present case illustrates several key diagnostic features that should prompt reconsideration of inflammatory arthritis in early childhood, particularly when clinical and laboratory findings are discordant with the expected profile of juvenile idiopathic arthritis. CACP syndrome is a well-documented mimicker of juvenile idiopathic arthritis (JIA), particularly the oligoarticular form, as both conditions may present with joint swelling in early childhood accompanied by preserved joint function and minimal pain [1,2]. This clinical overlap frequently leads to initial misclassification as JIA. However, in contrast to inflammatory arthritis, patients with CACP syndrome typically exhibit normal inflammatory markers and lack systemic features, key elements that should raise suspicion of an alternative, non-inflammatory diagnosis [1,9].

### 3.2. Therapeutic Approaches and Clinical Management

At present, there is no disease-specific approved therapy and no standardized surveillance protocol for patients with CACP syndrome. Management remains essentially supportive and multidisciplinary, guided by the clinical manifestations and severity of musculoskeletal and extraskeletal involvement [2,11,12].

Because CACP is a non-inflammatory arthropathy caused by PRG4/lubricin deficiency, conventional anti-inflammatory and immunosuppressive therapies are not expected to modify the underlying disease mechanism. In published series and case reports, patients frequently showed absent or minimal benefit from corticosteroids, methotrexate, and biologic agents, despite initially being treated as juvenile idiopathic arthritis. Once the diagnosis of CACP is established, unnecessary exposure to these therapies should therefore be avoided [11,12,13].

Current management should focus on supportive care. Physiotherapy and functional rehabilitation are central, with the aim of maintaining joint range of motion, preserving function, reducing secondary contractures, and limiting disability over time. Although evidence is based mainly on case reports and small series, physiotherapy has been associated with improvement in mobility and pain in some patients and is generally regarded as a core component of care [2,11].

Orthopedic follow-up is also important, particularly for progressive deformities such as camptodactyly and coxa vara. Conservative measures, including splinting, may be useful in selected cases of hand involvement, while surgical intervention may be considered when deformity becomes functionally significant or when hip disease progresses. However, orthopedic treatment is individualized, since no standardized surgical algorithm has been established for CACP [2,11].

Given the potential for pericardial involvement, patients should undergo cardiologic surveillance, especially when symptoms or imaging findings raise concern for pericardial effusion. Drainage procedures may be required in cases of hemodynamic compromise or clinically significant effusion. Families should be educated regarding warning symptoms suggestive of pericardial disease, such as pleuritic chest pain, dyspnea, fatigue, or unexplained fever, so that medical assessment can be sought promptly [11,12].

### 3.3. Emerging Therapeutic Perspectives

The growing understanding of CACP pathophysiology has made lubricin restoration an especially attractive therapeutic concept. *PRG4* encodes lubricin, a key boundary lubricant that protects cartilage surfaces and helps regulate synovial homeostasis; loss of lubricin function is central to CACP pathogenesis [8,14].

On this basis, recombinant lubricin replacement has emerged as a plausible future strategy. Preclinical work in animal models has shown that restoration of lubricin can prevent or attenuate structural joint damage associated with congenital lubricin deficiency, supporting the biological rationale for replacement-based treatment. However, this approach remains experimental and has not yet been established as a clinical therapy for CACP in humans [15,16,17].

A second promising direction is *PRG4* gene therapy. Intra-articular adeno-associated virus (AAV)-mediated *PRG4* delivery has shown encouraging preclinical results in joint disease models, demonstrating the feasibility of local lubricin restoration. Even so, these studies are still preclinical and were not conducted specifically as established human treatment for CACP syndrome [15,18].

More broadly, experimental studies have also explored intra-articular PRG4-based biologic supplementation and related strategies aimed at restoring boundary lubrication and reducing cartilage damage. These findings reinforce the concept that future treatment of CACP may evolve from symptomatic support toward mechanism-based therapy, although this transition has not yet occurred in routine clinical practice [19,20,21].

Overall, current care for CACP remains supportive, but lubricin-targeted approaches represent the most biologically coherent emerging therapeutic avenue. At present, these strategies should still be regarded as investigational [17,18].

### 3.4. Diagnostic Pitfalls in Pediatric Practice

The clinical resemblance between CACP syndrome and JIA contributes to a major diagnostic pitfall in pediatric rheumatology: the assumption that persistent joint effusion necessarily reflects inflammatory arthritis. This assumption may delay accurate diagnosis and expose patients to unnecessary immunosuppressive therapies. In particular, persistence of joint swelling despite normal inflammatory parameters and absence of clinical response to methotrexate or biologic agents should prompt careful diagnostic reassessment and consideration of CACP syndrome [1,2].

Misdiagnosis of CACP syndrome as juvenile idiopathic arthritis is well documented, particularly in early disease stages due to overlapping clinical features [1,9]. Although precise estimates vary, a proportion of pediatric patients initially diagnosed with JIA may later be reclassified as having non-inflammatory arthropathies. These conditions, while less frequent, are clinically significant as they require fundamentally different management strategies [5,10]. The rarity of CACP syndrome and limited awareness among clinicians contribute to diagnostic delays and misclassification.

### 3.5. Role of Imaging

In this context, imaging becomes a critical tool for refining the differential diagnosis. Radiological findings play a central role in distinguishing CACP syndrome from inflammatory arthropathies. The absence of erosive changes, together with metaphyseal widening and the characteristic Erlenmeyer flask-like deformity, strongly supports a non-inflammatory etiology [2]. Complementary imaging with ultrasound typically reveals joint effusion without Doppler signal or other features of active synovitis, further helping to differentiate CACP syndrome from JIA [22]. Recent studies have highlighted the role of musculoskeletal ultrasound in differentiating CACP syndrome from inflammatory arthropathies, particularly in pediatric patients, supporting earlier and more accurate diagnosis [23].

### 3.6. Synovial Fluid and Pathology

When diagnostic uncertainty persists, synovial fluid analysis and histopathological evaluation provide additional supportive evidence. Synovial fluid in CACP syndrome usually demonstrates low cellularity with mononuclear predominance, in contrast to the inflammatory profiles seen in JIA [22]. Histopathological studies further corroborate this distinction, revealing synovial hyperplasia in the absence of inflammatory infiltrates, findings that are consistent with lubricin deficiency rather than immune-mediated synovitis [22].

### 3.7. Genotype–Phenotype Considerations

Finally, integration of clinical, imaging, and pathological findings underscores the importance of genetic confirmation. Genetic studies have demonstrated marked phenotypic variability among patients with CACP syndrome, even within the same family, highlighting the limitations of clinical criteria alone [5,6,10]. Identification of pathogenic variants in *PRG4* establishes a definitive diagnosis and provides essential information for prognosis, family screening, and genetic counseling.

Both variants identified in this patient are frameshift alterations predicted to generate truncated PRG4 proteins and consequent loss-of-function of lubricin. Truncating variants represent one of the most common molecular mechanisms underlying CACP syndrome, leading to reduced or absent lubricin secretion within the synovial environment. Loss of lubricin disrupts the boundary lubrication layer of articular cartilage and contributes to progressive synovial hyperplasia and chronic non-inflammatory joint effusion, which constitute the hallmark features of the disease.

### 3.8. Molecular Basis of PRG4-Related Arthropathy

Beyond its clinical relevance, CACP syndrome provides an important model for understanding the molecular mechanisms underlying joint homeostasis. The disease results from pathogenic variants in the *PRG4* gene, which encodes lubricin, a large mucin-like glycoprotein secreted by synovial fibroblasts and superficial zone chondrocytes. Lubricin plays a central role in boundary lubrication of articular cartilage, reducing friction between opposing cartilage surfaces and protecting joint tissues from mechanical damage.

Loss-of-function variants in PRG4, such as the truncating variants identified in our patient, lead to reduced or absent lubricin production. This deficiency disrupts the normal lubricating layer at the cartilage surface and alters synovial fluid rheology. Experimental studies have demonstrated that lubricin deficiency results in increased mechanical stress at the cartilage interface, progressive synovial hyperplasia, and accumulation of non-inflammatory joint effusion. These mechanisms explain why patients with CACP syndrome develop chronic joint swelling despite the absence of inflammatory pathways typically involved in autoimmune arthritis.

From a pathophysiological perspective, the distinction between inflammatory and mechanical synovial pathology is critical. In inflammatory arthritis such as JIA, synovial hypertrophy is driven by immune-mediated processes involving cytokines and cellular infiltration. In contrast, in CACP syndrome synovial proliferation appears to represent a compensatory response to altered joint lubrication and mechanical stress. Histopathological analyses consistently demonstrate synovial hyperplasia with minimal inflammatory infiltrates, supporting a fundamentally different biological mechanism.

The molecular diagnosis of PRG4 deficiency therefore has direct clinical implications. Identification of pathogenic variants not only confirms the non-inflammatory nature of the disease but also prevents unnecessary exposure to immunosuppressive therapies. In addition, genetic confirmation enables appropriate family counseling and contributes to the growing understanding of lubricin biology and its role in joint physiology.

Finally, this case highlights how rare monogenic disorders can illuminate key molecular pathways involved in common musculoskeletal diseases. Increasing recognition of *PRG4*-related arthropathy emphasizes the importance of integrating genomic diagnostics into the evaluation of atypical pediatric rheumatologic presentations, particularly when clinical and laboratory features do not support an inflammatory etiology.

## 4. Conclusions

Early recognition of CACP syndrome is essential to avoid misdiagnosis as juvenile idiopathic arthritis and to prevent unnecessary and potentially harmful exposure to immunosuppressive therapies. An integrated diagnostic approach combining clinical assessment, laboratory investigations, imaging findings and molecular genetic analysis enables accurate diagnosis and supports appropriate, individualized patient management [1,7]. In this context, close collaboration between pediatric rheumatologists, geneticists and radiologists is crucial for the timely identification of rare non-inflammatory arthropathies.

Rare monogenic disorders such as CACP syndrome also provide valuable models for understanding fundamental mechanisms of joint biology. In particular, PRG4 deficiency highlights the essential role of lubricin in maintaining synovial joint homeostasis. Increasing integration of genomic diagnostics into pediatric rheumatology will likely improve recognition of non-inflammatory arthropathies and refine clinical decision-making when classical inflammatory markers and therapeutic responses are absent. These findings illustrate how molecular diagnosis can directly influence clinical decision-making and prevent inappropriate therapeutic interventions in pediatric rheumatology.

## Data Availability

The original contributions presented in this study are included in the article. Further inquiries can be directed to the corresponding author.
